# Targeting Prostate Cancer Using Bispecific T-Cell Engagers against Prostate-Specific Membrane Antigen

**DOI:** 10.1021/acsptsci.3c00159

**Published:** 2023-10-06

**Authors:** Gargi Das, Jakub Ptacek, Barbora Havlinova, Jana Nedvedova, Cyril Barinka, Zora Novakova

**Affiliations:** † Laboratory of Structural Biology, Institute of Biotechnology of the Czech Academy of Sciences, BIOCEV, Prumyslova 595, 252 50 Vestec, Czech Republic; ‡ Department of Cell Biology, Faculty of Science, Charles University, 128 00 Prague, Czech Republic

**Keywords:** prostate cancer, glutamate carboxypeptidase II, antibody engineering, bispecific T-cell engager (BiTE), immunotherapy, prostate-specific membrane antigen (PSMA)

## Abstract

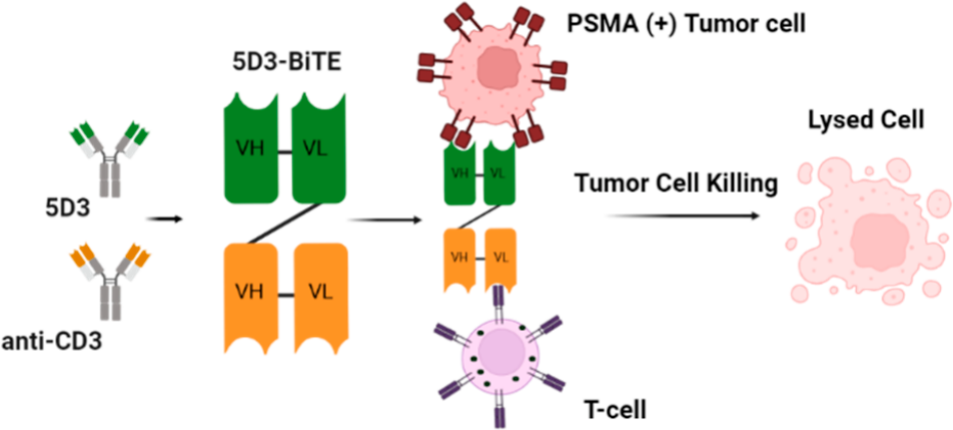

Prostate cancer (PCa) tops the list of cancer-related deaths in men worldwide. Prostate-specific membrane antigen (PSMA) is currently the most prominent PCa biomarker, as its expression levels are robustly enhanced in advanced stages of PCa. As such, PSMA targeting is highly efficient in PCa imaging as well as therapy. For the latter, PSMA-positive tumors can be targeted directly by using small molecules or macromolecules with cytotoxic payloads or indirectly by engaging the immune system of the host. Here we describe the engineering, expression, purification, and biological characterization of bispecific T-cell engagers (BiTEs) that enable targeting PSMA-positive tumor cells by host T lymphocytes. To this end, we designed the 5D3-αCD3 BiTE as a fusion of single-chain fragments of PSMA-specific 5D3 and anti-CD3 antibodies. Detailed characterization of BiTE was performed by a combination of size-exclusion chromatography, differential scanning fluorimetry, and flow cytometry. Expressed in insect cells, BiTE was purified in monodisperse form and retained thermal stability of both functional parts and nanomolar affinity to respective antigens. 5D3-αCD3’s efficiency and specificity were further evaluated *in vitro* using PCa-derived cell lines together with peripheral blood mononuclear cells isolated from human blood. Our data revealed that T-cells engaged via 5D3-αCD3 can efficiently eliminate tumor cells already at an 8 pM BiTE concentration in a highly specific manner. Overall, the data presented here demonstrate that the 5D3-αCD3 BiTE is a candidate molecule of high potential for further development of immunotherapeutic modalities for PCa treatment.

Prostate cancer (PCa) is the second leading cause of cancer-related death in men.^1^ Unlike localized PCa, which can often be treated successfully with surgery or radiation, there is currently no cure for metastatic PCa, and the development of new treatment modalities is thus critical for the successful management of metastatic PCa.

Prostate-specific membrane antigen (PSMA, EC 3.4.17.21), also known as glutamate carboxypeptidase II, N-acetylated-alpha-linked acidic dipeptidase, and folate hydrolase, is a class II transmembrane protein expressed in various tissues, including the prostate gland, Schwann cells of the central nervous system, duodenal brush border cells of the small intestine, and the proximal renal tubules of the kidney.^2−6^ This clinically well-established marker of PCa^7^ shows progressive upregulation throughout almost all stages of PCa, from prostatic neoplasia to the metastatic form of castration-resistant prostate cancer (mCRPC).^8−11^ The highly specific and abundant expression makes PSMA an excellent target for PCa imaging and therapy.

Immuno-oncology is a rapidly developing field with high promise for the development of effective cancer therapies.^12,13^ Accordingly, various immunotherapy approaches and tools intended for clinical treatments of PCa are currently being intensively studied, with the interest focused mainly on PSMA-specific monoclonal antibodies.^14^ These antibodies might be preferred over small molecules in therapy of PCa since the majority of small molecules targeting PSMA also bind glutamate carboxypeptidase III, a poorly characterized PSMA paralog.^15,16^ The ^177^Lu-DOTA-J591 conjugate can serve as an example of a PSMA-specific antibody evaluated for radiotherapy of mCRPC. While treatment with the radiopharmaceutical resulted in a significant decrease in prostate-specific antigen (PSA) in 60% of the patients, myelotoxicity was a major undesired side effect, limiting its potential therapeutic value.^17^ Antibody–drug conjugate (ADC) therapy is yet another example of an immuno-oncology approach targeting PSMA that advantageously exploits PSMA internalization upon binding of an ADC conjugate.^18,19^ PSMA-specific antibodies conjugated to microtubule-targeting toxins entered clinical studies; however, the efficient anti-tumor effects of these drug conjugates were accompanied by adverse effects, including peripheral neuropathy and neutropenia.^20^

In various cancer treatments, antibodies are also used as mediators of interactions between cancer cells and host immune cells. Such interactions can specifically attract immune cells toward targeted cancer cells, resulting in directed attack on a tumor. Engagement between immune and tumor cells could be controlled by distinctly engineered molecules, including chimeric antigen T-cell receptors (CAR Ts) and bispecific T-cell engagers (BiTEs),^21−23^ which are designed to selectively recruit T-cells to the tumor site. The focus on T-cells is rationalized by their high abundance in peripheral blood as well as their ability to kill target cells by secreted perforins and granzymes or by inducing programmed cell death via Fas ligand signaling.^21,22,24−27^ BiTEs can be a preferred choice over CAR Ts due to their significantly lower production costs, as they do not require the complex and expensive manufacturing process necessary for CAR T-cells. A BiTE consists of two structurally independent parts, where one site specifically binds a T-cell receptor while the second site is derived from a binder molecule (antibody) to recognize a target antigen on the surface of a tumor cell.^24,28,29^ Simultaneous binding to the ε-chain of the T-cell receptor and the tumor-specific antigen enables BiTE to form an immunologic synapse between the cancer cell and the T lymphocyte to activate the latter.^30^ In response, the T-cell is activated, and the process is accompanied by enhanced surface presentation of CD69 and CD25, which are early and late activation markers, respectively.^31−33^ Additionally, BiTE technology brings the advantage of polyclonal T-cell activation independent of MHC (the major histocompatibility complex)-driven antigen presentation. Consequently, BiTEs can even target tumor cells that escape the immune surveillance through downregulation of MHC expression.^34^

Pasotuxizumab (AMG-212) is a first-generation PSMA-targeting BiTE designed for PCa therapy.^35^ While AMG-212 has shown some promise in a Phase I clinical trial, 90% of patients developed drug-neutralizing antibodies,^35,36^ thus necessitating the development of AMG-160, an advanced derivative of AMG-212, which is currently under active investigation. These results clearly show that the development of BiTEs for PCa therapy is a viable approach, and data presented in this report extend the findings in the literature. Here, we use our recently developed 5D3 antibody with high specificity and nanomolar affinity toward PSMA^37−39^ to engineer 5D3 BiTE derivatives that effectively mediate T-cell cytotoxicity toward PSMA-expressing cells in sub-nanomolar concentrations. Our data thus further illustrate the high potential of 5D3 BiTE as a lead molecule for the development of modalities for immunotherapy of prostate cancer.

## Results

### Design, Expression, and Purification of Recombinant Constructs

**Figure 1:**
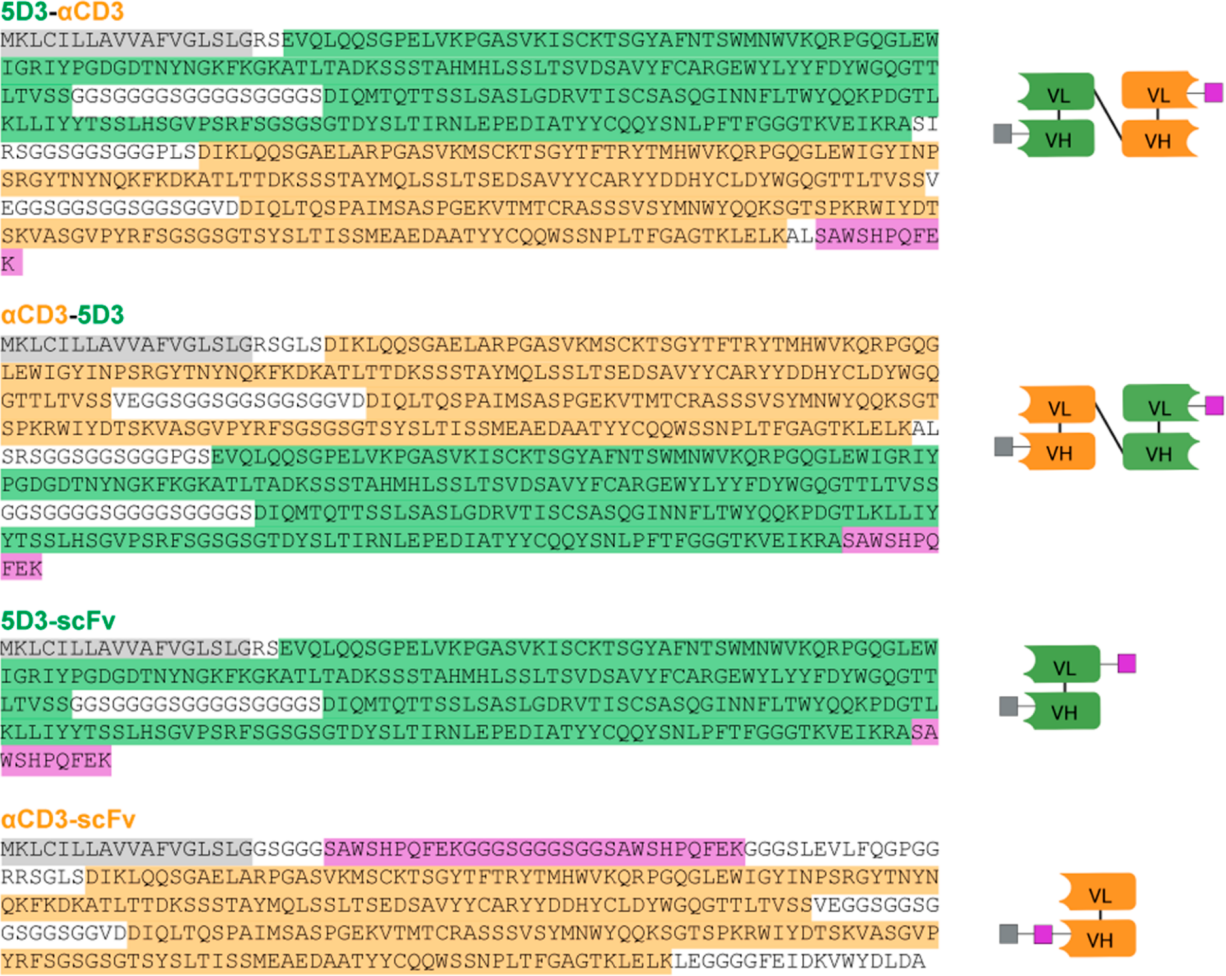
Scheme of 5D3 BiTE variants and individual single chains. Amino acid sequences of 5D3-αCD3, αCD3-5D3, 5D3-scFv, and αCD3-scFv are shown on the left, and schematic representations of the corresponding proteins are shown on the right; VH and VL refer to variable heavy and light domains, respectively. The N-terminal BiP secretion signal sequence and affinity SA-Strep II/Twin-Strep tags are colored gray and purple, respectively. Antibody-variable domains of 5D3 and αCD3 are colored green and orange, respectively. Created with BioRender.com.

**Figure 2:**
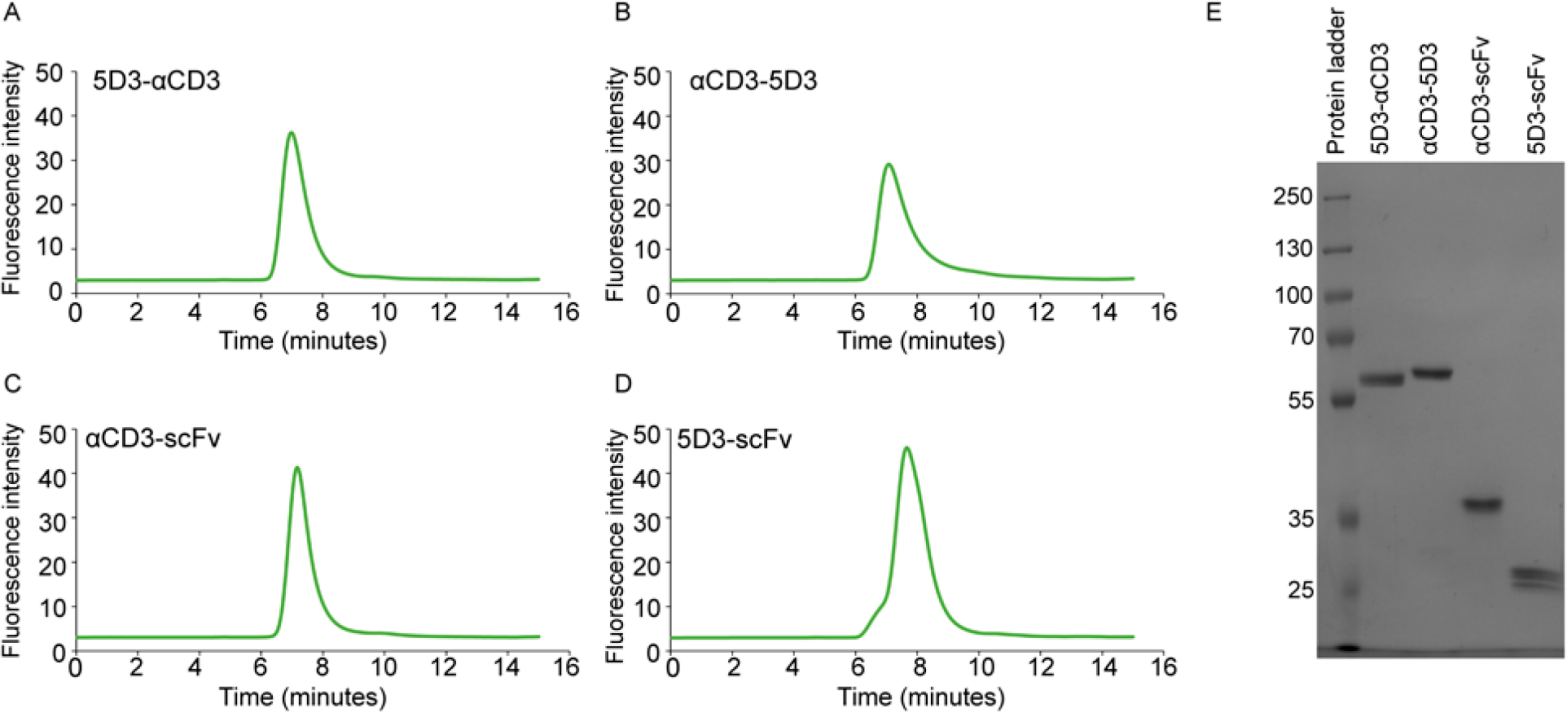
Analytical SEC of final protein preparations: 5D3-αCD3 (A), αCD3-5D3 (B), αCD3-scFv (C), and 5D3-scFv (D), revealing a homogeneous monomeric fraction. Protein purity >95% is documented by the Coomassie Brilliant Blue G-250-stained gel (E).

Two 5D3 BiTE variants were designed to test the effect of single-chain variable fragment (scFv) order on expression levels, purity, and stability (Figure 1). The sequence of 5D3-scFv originated from the previously published 5D3-scFv HL plasmid,^39^ whereas the αCD3-scFv sequence was derived from the αCD19/αCD3 Blinatumomab BiTE.^40,41^ In the fusion constructs, both scFvs were connected by flexible linkers of 16 and 17 amino acids for 5D3-αCD3 and αCD3-5D3, respectively. The fusions were inserted in the pMT expression vector, enabling secretion of overexpressed proteins into cultivation media directed by a cleavable BiP leader sequence at the N-terminus of a fusion. The SA-strep tag II was inserted at the C-terminus of fusion proteins to facilitate purification steps. The αCD3-scFv construct was designed with the N-terminal Twin-strep tag. All constructs were heterologously expressed in insect Schneider’s S2 cells and purified from culture media by a combination of affinity and size exclusion chromatography (SEC). 5D3-αCD3, αCD3-5D3, and αCD3-scFv constructs were produced in a predominantly monomeric form with yields of 1.6, 1.8, and 3.5 mg/L, respectively, and with purity >95% as estimated by Coomassie-stained SDS-PAGE (Figures 2 and S1).

### Thermal Stability

**Figure 3:**
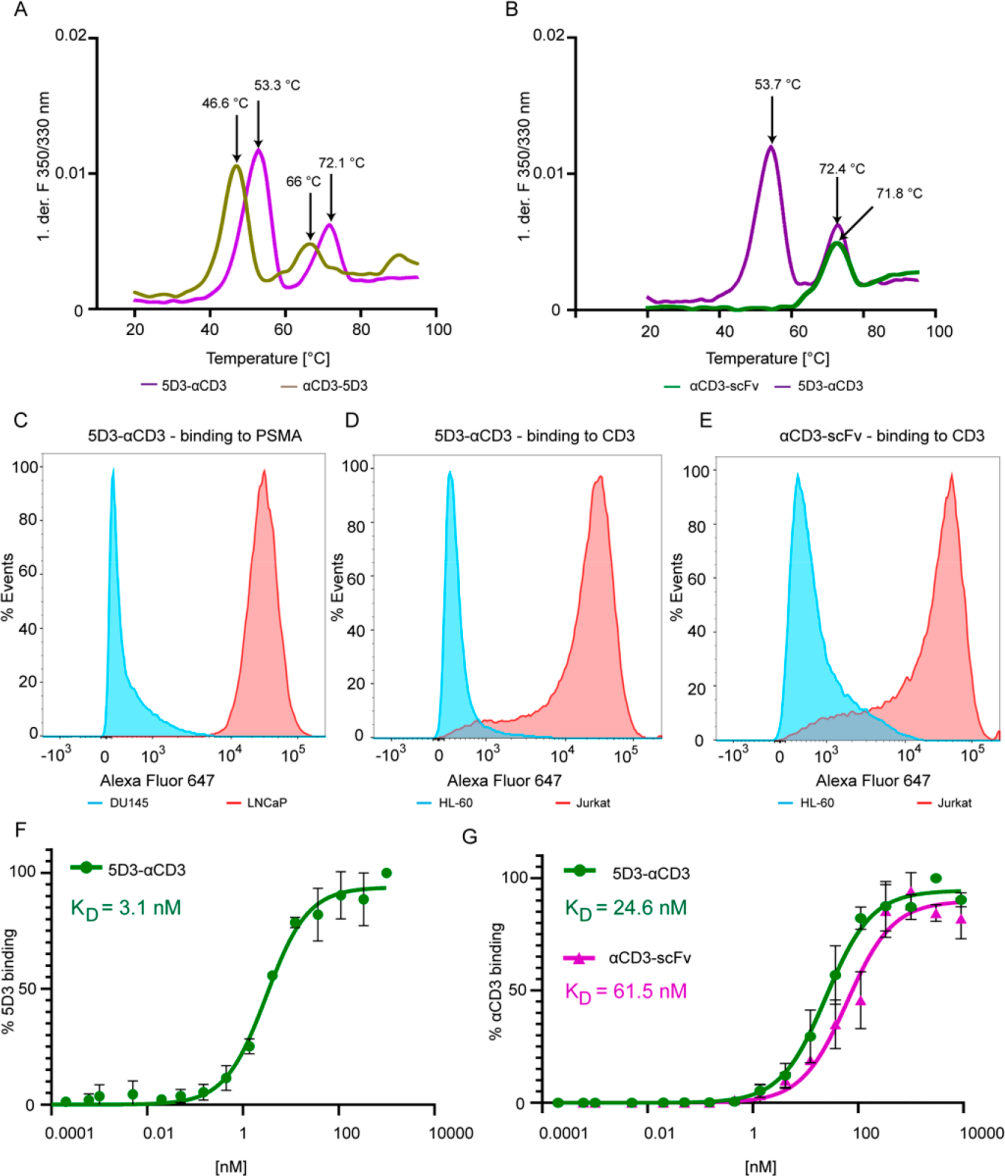
Characterization of purified BiTE variants. (A, B) Thermal stability determined by nanoDSF. The first derivatives of intrinsic fluorescence (ratio 350/330 nm) of 5D3 BiTE variants (A) and αCD3-scFv (B) are shown. (C–E) Target specificity of individual arms of the 5D3-αCD3 BiTE determined using PSMA-positive LNCaP cells (the 5D3 arm at 10 nM concentration; panel C) and CD3-positive Jurkat cells (the αCD3 arm at 100 nM concentration; panel D) using flow cytometry. αCD3-scFv (1000 nM) was included as a specific control of the αCD3 arm (panel E). DU145 and HL-60 cells served as PSMA- and CD3-negative controls, respectively. Approximately 50,000 live cells were used to generate individual histograms. (F, G) Affinities (*K*_D_) of 5D3 (F) and αCD3 (G) arms of the BiTE, and αCD3 scFv (G) determined by flow cytometry using LNCaP and Jurkat cells, respectively. DU145 and HL-60 cells were used as negative controls.

The thermal stability of purified 5D3 BiTEs was assessed by using nano differential scanning fluorimetry (nanoDSF). The thermal profile of 5D3-αCD3 revealed two peaks, corresponding to melting temperatures (*T*_m_) of 53.3 and 72.1 °C, while αCD3-5D3 showed *T*_m_ values of 46.6 and 66 °C (Figure 3A). By comparing the temperature profiles of our fusions with the melting temperatures of isolated 5D3-scFv (*T*_m_ = 53.4 °C; ref (39)) and αCD3-scFv (*T*_m_ = 71.8 °C, Figure 3B), we found an excellent correlation in the case of the 5D3-αCD3 BiTE, indicating retained stability of individual domains in the fusion. On the other hand, αCD3-5D3 showed significantly decreased *T*_m_ by 6 °C for both functional parts, suggesting compromised stability of the αCD3-5D3 fusion. Consequently, only the more stable 5D3-αCD3 BiTE was used in subsequent experiments.

### Specificity and Binding Affinity of 5D3-αCD3

The specificity of 5D3-αCD3 toward the respective target antigens was determined by flow cytometry. For the PSMA-targeting arm, PSMA-positive LNCaP, HEK 293T/PSMA, and PC-3 PIP cells together with PSMA-negative controls (DU145, HEK 293T/17, and PC-3) were used. Similarly, the specificity of the αCD3 arm was evaluated using CD3-positive Jurkat cells together with CD3-negative HL-60, Raji, and U-937 controls (Figures 3C,D and S2). In line with predicted specificity, markedly increased fluorescence intensity was observed only for PSMA- and CD3-positive cell lines when compared to matching PSMA- and CD3-negative controls, demonstrating highly specific recognition of target antigens on the surface of live cells by 5D3-αCD3. These staining patterns were virtually identical when isolated αCD3-scFv and 5D3-scFv fragments were used in an identical experimental setup (Figure 3E and ref (39)).

Furthermore, the specificity of the BiTE against subsets of human immune cells from the peripheral blood mononuclear cells (PBMCs) pool was also determined by flow cytometry using double staining by 500 nM BiTE in combination with antibodies targeting CD3, CD14, CD19, and CD56 surface antigens as specific markers of T-cells, monocytes, B-cells, and NK-cells, respectively. As only the CD3-positive population, corresponding to T-cells, revealed binding of 5D3-αCD3 (as revealed by double-staining positivity in Figure S3), these findings show that the BiTE binding is limited to T-cells exclusively.

In the next step, we determined the affinity of individual arms of the 5D3-αCD3 BiTE using LNCaP and Jurkat cell lines. Here, 3-fold dilution series of the BiTE ranging from 9 μM to 2 pM were incubated with target cells, and cell surface binding was visualized by subsequent incubation with the mixture of the anti-Strep tag antibody and a fluorescently labeled secondary antibody using flow cytometry. Experimental data were fitted using a non-linear regression algorithm, and apparent dissociation constants (*K*_D_) were calculated to be 3.1 and 24.6 nM for the 5D3 and αCD3 arms, respectively (Figure 3F,G). For comparison, *K*_D_ values of isolated 5D3-scFv and αCD3-scFv fragments were determined to be 1.5 and 61.5 nM, respectively (Figure 3G and ref (39)).

### T-Cell Activation by 5D3-αCD3

**Figure 4:**
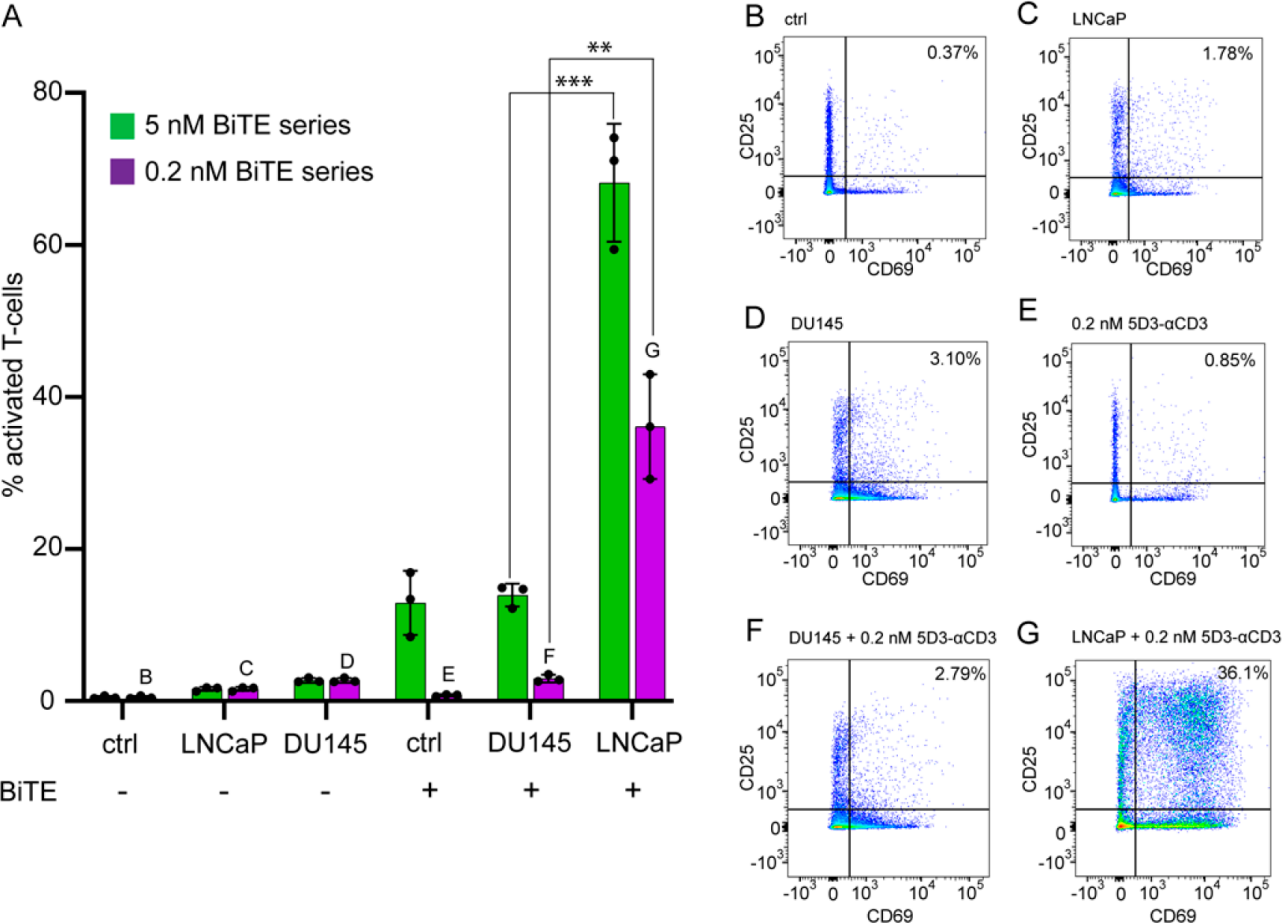
Activation of T-cells mediated by 5D3-αCD3. Human PBMCs (ctrl) were co-cultured with LNCaP or DU145 cells in the presence or absence of 5 or 0.2 nM 5D3-αCD3, as shown in panel A in green and purple, respectively. Expression levels of CD25/CD69 surface activation markers were determined by flow cytometry in the CD3-positive cell population. (A) Percentage of CD69/CD25 double-positive cells in the CD3+ T-cell population originated from isolated PBMCs. Statistical analysis was performed by non-parametric Student’s *t* test (*P*-value <0.01 (**), <0.001 (***)). (B–G) Scatter plots analyzing CD25 (vertical axis) and CD69 (horizontal axis) expression levels in isolated PBMCs (B), PBMCs co-cultured with LNCaP cells (C), PBMCs co-cultured with DU145 cells (D), PBMCs + 0.2 nM 5D3-αCD3 (E), PBMC/DU145 co-cultures + 0.2 nM 5D3-αCD3 (F), and PBMC/LNCaP co-cultures + 0.2 nM 5D3-αCD3 (G). The percentage of CD25/CD69 double-positive T-cells is shown inside individual plots.

To evaluate whether the 5D3-αCD3 BiTE can specifically activate T-cells in the presence of target PSMA-positive cancer cells, isolated human PBMCs were co-cultured with LNCaP cells (or DU145 controls) at the optimized effector-to-target ratio (E:T) of 3:1^42^ in the medium supplemented with 0.2 nM and 5 nM BiTE. Following 48-h incubation, the activation of the CD3-positive T-cell population in co-cultures was analyzed by flow cytometry using combinations of antibodies against CD25 and CD69, which represent early and late T-cell surface activation markers, respectively (Figure 4). In PBMC/LNCaP co-cultures in the presence of the 5D3-αCD3 BiTE, the surface expression of both activation markers was increased significantly, reaching 68.2% and 36.1% of CD25/CD69 double-positive CD3+ T-cells for 5D3-αCD3 BiTE concentrations of 5 and 0.2 nM, respectively (Figure 4A). On the other hand, no significant T-cell activation was observed in the absence of the BiTE or in PBMC/DU145 co-cultures. These data clearly show that 5D3-αCD3 can mediate PSMA-directed T-cell activation at sub-nanomolar concentrations *in vitro*. Of note, low levels of activated T-cells were also observed for 5 nM BiTE concentrations in the absence of PSMA-positive cells.

### 5D3-αCD3-Mediated Cytotoxicity

PSMA-targeted immunotoxicity mediated by 5D3-αCD3 was determined by quantifying the metabolic activity of live cells using the MTT cell viability assay. We first determined that, in the absence of PBMCs, 5D3-αCD3 alone does not negatively influence the viability of LNCaP or DU145 target cells (Figure S4). At the same time, in the absence of 5D3-αCD3, the co-incubation of target cells and effector PBMCs (at the optimized E:T ratio of 3:1, see below) led to a slight decrease in target cell viability, irrespective of the presence of PSMA expression at their surface. The latter findings suggest weak non-specific activation of isolated PBMCs under our experimental conditions.

**Figure 5:**
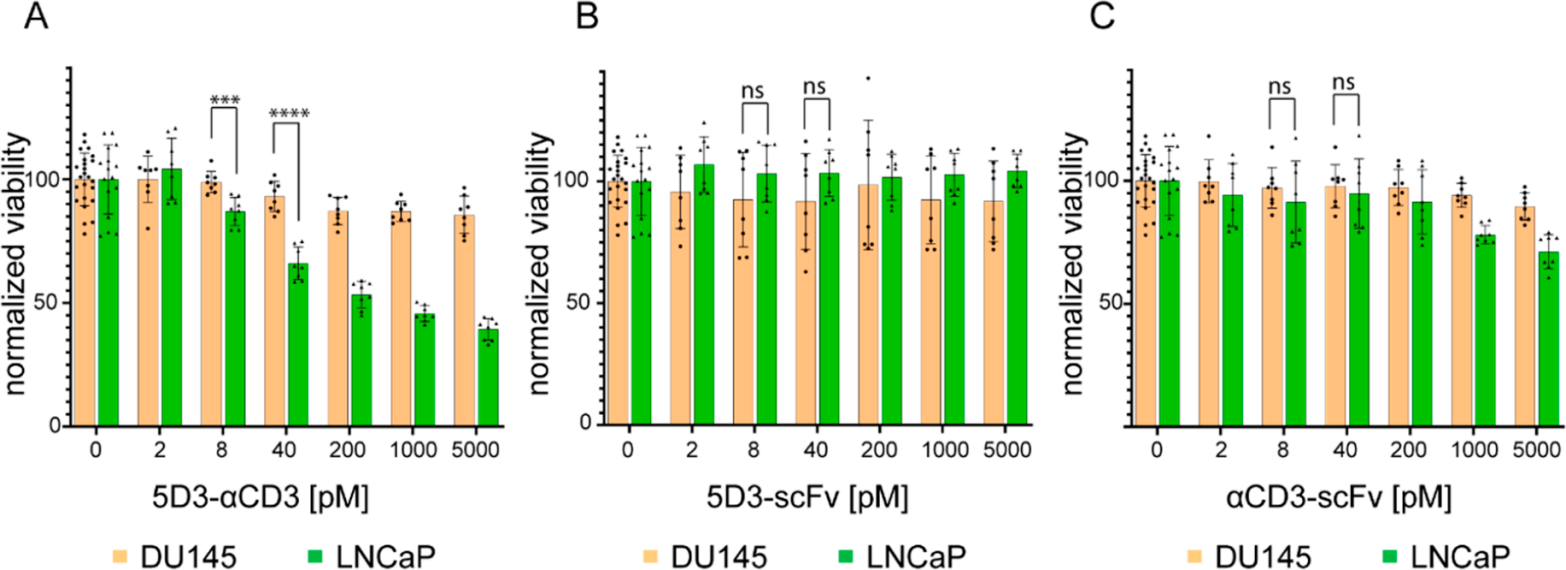
5D3-αCD3-mediated cytotoxicity. PBMCs were co-cultured with DU145 (orange) and LNCaP (green) cell lines in the presence of dilution series of 5D3-αCD3 (A), 5D3-scFv (B), and αCD3-scFv (C). Cell viability was determined by the MTT assay following 48-h incubation. Statistical analysis was performed by non-parametric Student’s *t* test (ns = non-significant, *P*-values <0.001 (***) and <0.0001 (****)).

To optimize assay conditions, we then tested several E:T ratios, and our data revealed that the 3-fold excess of effectors has sufficient potential for significant killing of the target cells in comparison to the control groups (Figure S5). Next, PBMCs were co-cultured with target cells at the E:T ratio of 3:1 in the presence of a dilution series of the BiTE, and the cell viability was quantified using the MTT assay following 48-h incubation (Figure 5). When LNCaP cells were co-cultured with PBMCs in the presence of 5D3-αCD3, we observed a marked decrease in cell viability in a concentration-dependent manner (Figure 5A). The specific killing of PSMA-positive cells was detected already at a BiTE concentration as low as 8 pM, manifested by a 12% decrease in cell viability. Killing efficacy was even more pronounced at higher 5D3-αCD3 concentrations, reaching a 46% decrease in cell viability in the presence of 5 nM BiTE. On the other hand, the viability of the PSMA-negative DU145 cells co-cultured with PBMCs under identical conditions was not diminished to a significant degree at any 5D3-αCD3 concentration tested (Figure 5A). Additionally, the specificity and efficacy of the fusion BiTE were verified by testing individual isolated scFvs, as neither 5D3-scFv nor αCD3-scFv significantly affected cell viability under any conditions tested (Figure 5B,C). Overall, these results confirmed that 5D3-αCD3 is highly specific and efficient in mediating killing of PSMA-positive targets via cytotoxic action of activated T lymphocytes.

## Discussion

Immunotherapy has made remarkable progress as a therapeutic strategy targeting PSMA-positive PCa, primarily focused on T-cell-directed cytotoxicity.^19,36,43−48^ Despite these advancements, immunotherapy of PCa is still in early developmental stages, mostly due to the significant challenges posed by the heterogeneity of the tumor microenvironment. Consequently, intensive development of new immunotherapy treatments and tools is still warranted.

We have successfully produced PSMA-specific BiTEs using a heterologous S2 insect cell expression system.^39,49,50^ The decision to use BiTEs as mediators of T-cell cytotoxicity was rationalized based on several advantageous features of BiTEs over CAR T-cells. While both strategies share similar mechanisms of action, BiTE technology is simpler compared to the production of CAR T-cells, and undesired effects are also not so prominent. The efficacy of BiTEs treatment is exclusively influenced by the spectrum of T-cells present in host tissues at the time of BiTE administration. In contrast, the efficiency of CAR T-cell technology depends strongly on the composition and sufficient numbers of circulating host T-cells available for CAR engineering during leukapheresis. Moreover, the efficiency of CAR T-cell therapy could be compromised by various disease-related factors that might manifest after CAR T-cell infusion. Notably, CAR T-cells have shown remarkable expansion in hematologic cancers but reveal very low efficacy against solid cancers. In contrast, BiTE technology, which does not rely on expansion of imported T-cells, offers superior potential for treatment of solid tumors such as PCa.^21,51^

It shall be noted that several PSMA-targeting BiTEs, including AMG-212, AMG-160, MOR-209, CC-1, and JNJ-081, have been tested in preclinical settings and/or entered clinical trials.^52−55^ While these drugs showed positive effects in targeting PCa, (pre)clinical data also highlighted challenges and adverse effects that need to be addressed and mitigated to develop safe and efficacious immunotherapeutics against PCa. For example, the short half-life of BiTE constructs limits their overall therapeutic efficacy, requiring engineering of constructs with prolonged serum half-lives and increased stability that could be achieved, e.g., by incorporating an albumin-binding domain, Fc antibody fragments, or pasylation sequences.^56−61^ Administration of BiTEs is also often accompanied by the development of anti-drug antibody (ADA) responses, leading to lower efficacy and unacceptable systemic toxicity. In such cases, antibody humanization, different routes of administration, or combination therapies, e.g., with prophylactic dexamethasone, might be used to mitigate ADA responses.^52,62^ Cytokine release syndrome is yet another “typical” adverse effect associated with (not only) anti-PSMA BiTE administration that must be closely monitored during clinical trials.^54,63−65^

PSMA-specific 5D3-αCD3 demonstrates highly specific binding to its respective antigens, as no binding was observed for either PSMA-negative cell lines (Figures 3 and S2) or CD3-negative immune cells, including B-cell, monocyte, and NK-cell populations (Figure S3). It shall be noted, however, that our BiTE (and CD3-targeting BiTEs in general) will, in principle, activate both CD8^+^ cytotoxic and CD4^+^ helper T-cell populations and the “off-target” activation of CD4^+^ cells can be manifested differently *in vitro* and *in vivo*. As for *in vitro* settings, activation of CD4^+^ cells can lead to more pronounced cytotoxic effects, as these cells are capable of eliminating cancer cells through the granzyme perforin pathway.^31,66−68^ On the other hand, activation of CD4^+^ T-cells, including regulatory T-cells (T-regs), *in vivo* might be associated with suppression of immune responses and lower BiTE efficacy. In fact, poor prognosis and low survival rates have been reported for PCa patients with higher T-regs counts.^69^ Consequently, combination therapies including Treg depletion along with the BiTE treatment might be preferred for better personalized treatment.^70^ Overall, the above examples highlight challenges encountered in immunotherapy of solid tumors and accent the need for the development of novel molecules for the treatment of PCa.

While the 5D3 arm of the BiTE has affinity for PSMA comparable to that of the original 5D3-scFv,^39^ the αCD3-scFv arm surprisingly exhibits a significantly lower apparent *K*_d_ (24.6 nM) than that determined for the clinically approved BiTE Blinatumomab (260 nM).^71,72^ The difference can point toward more efficient folding and function of the αCD3 arm when present in combination with the 5D3 part.

It is interesting to note that, while *K*_D_ values for individual arms of the 5D3-αCD3 BiTE are in the low nanomolar range, biological effects are observed at sub-nanomolar concentrations. This phenomenon has already been observed for other bispecific molecules, including MOR209/ES414 that had *K*_D_ = 4 and 2 nM for the anti-PSMA and anti-CD3 arms, respectively.^53^ Another BiTE, Blinatumomab, revealed *K*_D_ = 1.49 and 260 nM for the anti-CD19 and anti-CD3 arms, respectively.^72^ In contrast to *K*_D_ values running in the nanomolar range, both drugs were reported to show significant killing efficiency already in the pico- to femtomolar concentration range. Moreover, in Blinatumomab application, the absence of cell clustering and the presence of cytolytic synapses were well documented, together with the ability of activated T-cells to kill the target cells in a serial manner.^71^

Pronounced cytotoxic effects of 5D3-αCD3 were observed even at the E:T ratio of 3:1 after 48-h co-cultivation. Interestingly, the killing efficiency of the BiTE at higher E:T ratios did not increase significantly, proving that an increased number of T-cells, while effective, is not a prerequisite. The data suggest that the limiting factor of the 5D3-αCD3 killing efficiency is the pool of BiTE molecules bound to target cells and not the number of T-cells available. We can thus hypothesize that the BiTE would be efficient also in patients harboring compromised numbers of functional T-cells. Interestingly, other studies show comparable killing efficiency with E:T ratios varying from 5:1 to 10:1.^36,47,57,73^ The 5D3-αCD3 molecule exhibited excellent efficacy in killing target cells, achieving 46% cell killing at 5 nM concentration (0.3 μg/mL) and E:T ratio of 3:1 after 48-h incubation. In comparison, one of the leading molecules, BiJ591 (BiTE constructed from the anti-PSMA J591 antibody), at a concentration 5 μg/mL, demonstrated 20% target cell killing efficiency with the same E:T ratio following 5-h incubation.^74^ Moreover, BiJ591 reached 50% killing efficiency only with an E:T ratio of 12:1. These results suggest that the performance of our BiTE in comparison to BiJ591 might be superior due to its low nanomolar affinity for PSMA, emphasizing its enhanced ability to effectively eliminate target cells.

Our results clearly document the high specificity of 5D3-αCD3. The BiTE activates T-cells only when associated with PSMA-positive targets, mitigating undesired off-target effects. The reported BiTE can benefit from the much higher affinity of the 5D3 arm toward PSMA (as compared, for example, with the J591 antibody^37^), and its strict specificity can contribute to the enhanced safety profile and ensure effective cancer cell eradication as a result of direct contacts between the target and effector T-cells.^75^ At the same time, it is clear that further optimization/engineering, such as 5D3 humanization, might be needed to improve the translatability of the reported 5D3 into the clinic, and only clinical trials could address its real-life scenario therapeutic potential.

## Conclusion

We have developed a novel 5D3-based BiTE that can mediate specific interactions between PSMA-positive prostate cancer cells and cytotoxic T-cells. Both functional parts of the BiTE have low-nanomolar affinity and high selectivity toward respective antigens. Additionally, sub-nanomolar concentrations of 5D3-αCD3 were highly efficient in killing PSMA-positive cells in picomolar concentrations *in vitro*. Given the encouraging *in vitro* performance, ensuing investigations including *in vivo* testing are warranted to evaluate the biomedical potential of the 5D3-αCD3 BiTE in more detail. Moreover, the molecule could be potentially involved in the studies of other solid tumors expressing the PSMA antigen.

## Methods

### Chemicals and Reagents

All chemicals and reagents were purchased from Sigma (Steinheim, Germany) unless specified otherwise. Restriction enzymes and ligase were purchased from New England Biolabs (Ipswich, MA, USA).

### Cells

LNCaP cells were kindly provided by Z. Hodny (IMG, Prague, Czech Republic), whereas PC-3 and PC-3 PIP cells were kindly provided by Dr. Warren Heston (Cleveland Clinic, Cleveland, OH, USA). DU145, HL-60, Raji, U-937, and Jurkat cell lines were obtained from the American Type Culture Collection (ATCC, USA). The HEK 293T/17 cell line overexpressing PSMA (HEK 293T/PSMA) was established in-house previously.^76^ HEK 293T/17 cells were cultivated in high-glucose DMEM media, whereas other cell lines and PBMCs were cultivated in RPMI-1640 media under a 5% CO_2_ atmosphere at 37 °C. Both media were supplemented with 10% fetal bovine serum (FBS) and 2 mM l-glutamine (Life Technologies, Thermo Fisher Scientific, Carlsbad, CA, USA). Penicillin 50 U/mL and streptomycin 50 μg/mL were used during PBMCs’ cultivation. Insect Schneider’s S2 cells (Invitrogen, Thermo Fisher Scientific, Carlsbad, CA, USA) were maintained at 26 °C in Insect-XPRESS media (Lonza, Basel, Switzerland) supplemented with 2 mM l-glutamine.

### Isolation of Peripheral Blood Mononuclear Cells

Buffy coats derived from the full blood of human donors were kindly provided by Dr. Petr Turek (Department of Transfusion Medicine and Blood Bank, Thomayer University Hospital, Prague, Czech Republic). The buffy coats were diluted in phosphate-buffered saline (PBS) at a 1:1 ratio, mixture was split to 35 mL aliquots and layered onto 15 mL aliquots of Ficoll Histopaque. Cells were centrifuged at 400*g* for 30 min with brakes switched off, and PBMCs were isolated as described previously.^77^ Cells were cryopreserved at a concentration of 6 × 10^6^ cells/mL in freezing media containing 20% dimethyl sulfoxide (DMSO) and 80% FBS and stored in liquid nitrogen. To pass the resting phase of PBMCs before experiment, cells were thawed and cultivated at a concentration of 2 × 10^6^ cells/mL in the cultivation medium (RPMI-1640, 10% FBS, 2 mM l-glutamine, 50 U/mL penicillin, and 50 μg/mL streptomycin) supplemented with 10 U/mL interleukin 2 overnight.^78^ The next day, PBMCs were washed in the cultivation medium and used in further experiments.

### Construction of Expression Vectors

To construct expression vectors of 5D3 BiTE variants, the 5D3-scFv gene (heavy-to-light chain orientation) was excised from the scFv HL plasmid as described by Novakova et al.^39^ and ligated into the pMT/BiP vector backbone using Blunt/TA Ligase Master Mix. The αCD3-scFv gene (heavy-to-light chain orientation) was commercially synthesized based on the αCD3-scFv amino acid sequence^40,41^ and amplified by PCR using gene-specific primers (Table S1). Amplified DNA was ligated into pMT/BiP/5D3-scFv vectors to prepare the desired pMT/BiP/5D3-αCD3 and pMT/BiP/αCD3-5D3 expression vectors, respectively. In parallel, an isolated αCD3-scFv fragment was cloned into the pMT/BiP vector. The final plasmid sequence was confirmed by using Sanger sequencing (GATC Biotech, Ebersberg, Germany).

### Stably Transfected S2 Cells

The expression plasmids were transfected in Drosophila Schneider’s S2 cells using Effectene transfection reagent (Qiagen, Hilden, Germany), along with the selection plasmid pCoBLAST (Invitrogen), as described previously.^39^ Transfected cultures were grown in the presence of 40 μg/mL blasticidine (InvivoGen, San Diego, CA, USA) for approximately 3 weeks until stable transfectants were selected.

### Expression and Purification of 5D3 BiTEs

Stably transfected S2 cells were expanded, and the expression of recombinant proteins was induced with 0.7 mM CuSO_4_. After 7 days of cultivation, the conditioned medium was harvested as described previously^39^ and concentrated approximately 20-fold using a 10 kDa ultrafiltration membrane fitted into a tangential flow filtration apparatus (Sartorius, Göttingen, Germany). 5D3 BiTEs were purified via Strep-Tactin XT affinity chromatography (IBA Lifesciences, Göttingen, Germany) with equilibration buffer comprising 50 mM Tris-HCl, 150 mM NaCl, and 10% glycerol, pH 8.0. Proteins were eluted by 5 mM d-biotin (VWR, Radnor, PA, USA) in the equilibration buffer and then further purified by SEC using a Superdex 200, 16/600 column (GE Healthcare Biosciences, Uppsala, Sweden) and an Enrich 650, 10/300 column (Bio-Rad Laboratories, Hercules, CA, USA) for the 5D3-αCD3 and αCD3-5D3 variants, respectively. PBS supplemented with 3% glycerol was used as a mobile phase in SEC runs operated by the NGC chromatography system (Bio-Rad Laboratories, Hercules, CA, USA). SEC fractions containing purified monodisperse fusion proteins were pooled, concentrated, and flash-frozen in liquid nitrogen for long-term storage at −80 °C. Analytical SEC of purified proteins (load equal to 7 μg) was performed using an Agilent Bio SEC-3, 100A column connected to an Agilent 1290 Infinity II liquid chromatography system (Santa Clara, CA, USA) that was equipped with an HPLC G7121B 1260 FLD detector (Agilent).

### Differential Scanning Fluorimetry (nanoDSF)—Thermal Stability

Purified constructs diluted in PBS to 0.3–0.5 mg/mL were loaded into glass capillaries, and protein unfolding was monitored using a Prometheus NT.48 fluorimeter (NanoTemper Technologies, München, Germany) with a temperature gradient from 25 to 95 °C at a rate set to 1 °C/min. *T*_m_ values of individual constructs were calculated from the ratio of fluorescence signals at 350 and 330 nm.

### Flow Cytometry

Cell harvesting, antibody staining, and signal detection were carried out according to Novakova et al.^39^ with minor modifications. Briefly, LNCaP, DU145, HEK 293T/17, HEK-293T/PSMA, PC-3, and PC-3 PIP cells were harvested by 0.025% Trypsin/0.01% EDTA/PBS, whereas Jurkat, HL-60, U-937, and Raji cells were harvested without trypsinization. Cells were mixed with BiTE in a total volume of 20 μL and incubated at 4 °C for 30 min. In experiments determining the affinity of BiTE, a 3-fold dilution series of 5D3-αCD3 (17 concentration points, final concentration range from 9 μM to 0.2 pM) was applied on cells. The cell-bound BiTE was detected by subsequent incubation with the mixture of anti-Strep tag mouse monoclonal antibody (1 μg/mL, Immo, IBA) and goat anti-mouse secondary antibody conjugated to Alexa Fluor 647 (4 μg/mL; Thermo Fisher Scientific). In the case of double staining of PBMCs, the cells were incubated with 500 nM BiTE. Following wash, individual samples were incubated with strepMAB-Immo DY-649 conjugate (f.c. 0.5 μg/mL; IBA) in combination with either anti-human CD14 mouse monoclonal antibody conjugated to PE Cyanine7 (dilution 1:100; BioLegend, San Diego, CA, USA), anti-human CD19 mouse monoclonal antibody conjugated to BV605 (dilution 1:100; BD Biosciences, San Jose, CA, USA), anti-human CD56 monoclonal antibody conjugated to APC/Cyanine7 (dilution 1:100; BioLegend), or anti-human CD3 monoclonal antibody (OKT3) conjugated to PerCP-Cyanine5.5 (f.c. 0.5 μg/mL; Invitrogen). The cells were washed 3–5 times after each incubation step. All dilution and washing steps were performed at 4 °C in PBS supplemented with 0.5% gelatin from coldwater fish skin. Cell viability was determined by Hoechst 33258 staining (final concentration of 0.1 μg/mL). The fluorescence intensity was measured using a BD LSRFortessa flow cytometer (BD Biosciences) and analyzed using FlowJo software (FlowJo, LLC, Ashland, OR, USA). *K*_D_ values of BiTE were calculated from the mode of fluorescence intensity in the GraphPad Prism software using a non-linear regression algorithm (GraphPad, San Diego CA, USA).

### Determination of T-Cell Activation

LNCaP or DU145 cells were mixed with PBMCs at an E:T ratio of 3:1, and then 5D3 BiTE at the desired concentration was added to the cell suspension to a total volume of 100 μL. Cell mixtures were further cultivated for 48 h. Post incubation, cells were harvested by centrifugation at 200*g* for 3 min and washed thoroughly three times with PBS/0.5% gelatin. Subsequently, anti-CD3 antibody (OKT3) conjugated to PerCP-Cyanine5.5, anti-human CD25 monoclonal antibody conjugated to PE (f.c. 2.5 μg/mL; BioLegend), and CyFlow anti-human CD69 mouse monoclonal antibody conjugated to APC (dilution 1:10; Sysmex, Kobe, Japan) were added to the cell suspension. Following 45-min incubation at 4 °C, cells were thoroughly washed, and Hoechst 33258 was added just before data acquisition using the BD LSRFortessa flow cytometer. Data processing was done using the FlowJo software, and results were visualized in the GraphPad Prism software.

### MTT Cytotoxicity Assay

Target cells at 0.5 × 10^4^/mL (total volume of 40 μL) were seeded in a 96-well flat-bottom plate and allowed to adhere to the plastic overnight. The next day, 50 μL of PBMCs was added (final E:T ratio of 3:1), followed by the addition of a 5-fold dilution series of 5D3-αCD3 (final concentration range from 0.3 pM to 5 nM) to a final volume of 100 μL. 5D3-scFv and αCD3-scFv were used as negative controls. The experiment was performed in phenol red-free cultivation media. After 48-h cultivation, 10 μL of the MTT reagent (3-[4,5-dimethylthiazol-2-yl]-2,5-diphenyltetrazolium bromide) was added to the cells at a final concentration of 0.45 mg/mL, and cells were further incubated at 37 °C for 90 min. Violet formazan crystals were then dissolved by the addition of 100 μL of the MTT solubilization buffer (14% sodium dodecyl sulfate, 40% dimethylformamide, and 2% acetic acid (Lachner, Neratovice, Czech Republic), pH 4.7) and incubated at room temperature for 3 h on a shaker set to 600 rpm. Absorbance at 570 nm was quantified using a Clariostar microplate reader (BMG Labtech, Ortenberg, Germany), and data were processed in the GraphPad Prism software.

## Supporting Information Available

Additional information about cloning primers (Table S1), size exclusion chromatography profiles (Figure S1), specificity of 5D3-αCD3 determined by flow cytometry (Figure S2 and S3), effect of 5D3-αCD3 on cell viability (Figure S4), and optimization of killing assay (Figure S5) (PDF)

## Abbreviations

ADA: anti-drug antibody
BiTE: bispecific T-cell engager
CAR: chimeric antigen receptor
DSF: differential scanning fluorimetry
E:T: effector-to-target ratio
FBS: fetal bovine serum
*K*_D_: dissociation constant
mCRPC: metastatic castration-resistant prostate cancer
MHC: major histocompatibility complex
PBMC: peripheral blood mononuclear cell
PCa: prostate cancer
PSA: prostate-specific antigen
PSMA: prostate-specific membrane antigen
scFv: single-chain variable fragment
SEC: size exclusion chromatography
*T*_m_: melting temperature
