# Supplementary material for: Targeting Prostate Cancer Using Bispecific T-Cell Engagers against Prostate-Specific Membrane Antigen
Source: ACS Pharmacol Transl Sci. 2023 Oct 6;6(11):1703–14. doi: 10.1021/acsptsci.3c00159 (PMC10644396; doi:10.1021/acsptsci.3c00159)
Supplement: Supplementary file 1 — pt3c00159_si_001.pdf [file pt3c00159_si_001.pdf]

SUPPORTING INFORMATION

Targeting prostate cancer using bispecific T-cell engagers against prostate-specific membrane antigen

Gargi Das[1,2], Jakub Ptacek[1], Barbora Havlinova[1], Jana Nedvedova[1], Cyril Barinka[1], and Zora Novakova[1]*

[1]Laboratory of Structural Biology, Institute of Biotechnology of the Czech Academy of Sciences, BIOCEV, Prumyslova 595, 252 50 Vestec, Czech Republic.

[2]Department of Cell Biology, Faculty of Science, Charles University Prague, Czech Republic.

*To whom correspondence should be addressed

Zora Nováková - Laboratory of Structural Biology, Institute of Biotechnology of the Czech Academy of Sciences, BIOCEV, Prumyslova 595, 252 50 Vestec, Czech Republic; Email: zora.novakova@ibt.cas.cz, Tel: +420 325 873 736

Table of Contents:

Table S1. List of primers for amplification of αCD3-scFv gene p. S-2

Figure S1. Purification of 5D3-BiTEs and αCD3-scFv expressed by S2 insect cells p. S-3

Figure S2. Specificity of 5D3-αCD3 determined by flow cytometry p. S-4

Figure S3. Specificity of 5D3-αCD3 against PBMC cell subsets determined by flow cytometry p. S-5

Figure S4. Effect of 5D3-αCD3 on the viability of target cells p. S-6

Figure S5. Optimization of the E:T ratio to promote specific killing efficiency of 5D3-αCD3 p. S-7

Table S1. List of primers of αCD3-scFv gene.

5D3 BiTE variant	Primers
5D3-αCD3	**Forward** - 5’- AAAAGATATCCGGTCCGGAGGTTCTGGTG-3’
	**Reverse** - 5’- TTTTCTCGAGTCATTATTTTTCGAACTGC-3’
αCD3-5D3	**Forward** - 5’- AAAAAGATCTGGGCTCAGCGACATCAAACTGC-3’
	**Reverse** - 5’- TTTTGGATCCCGGTCCGCCTCCACTTC-3’

Figure S1:

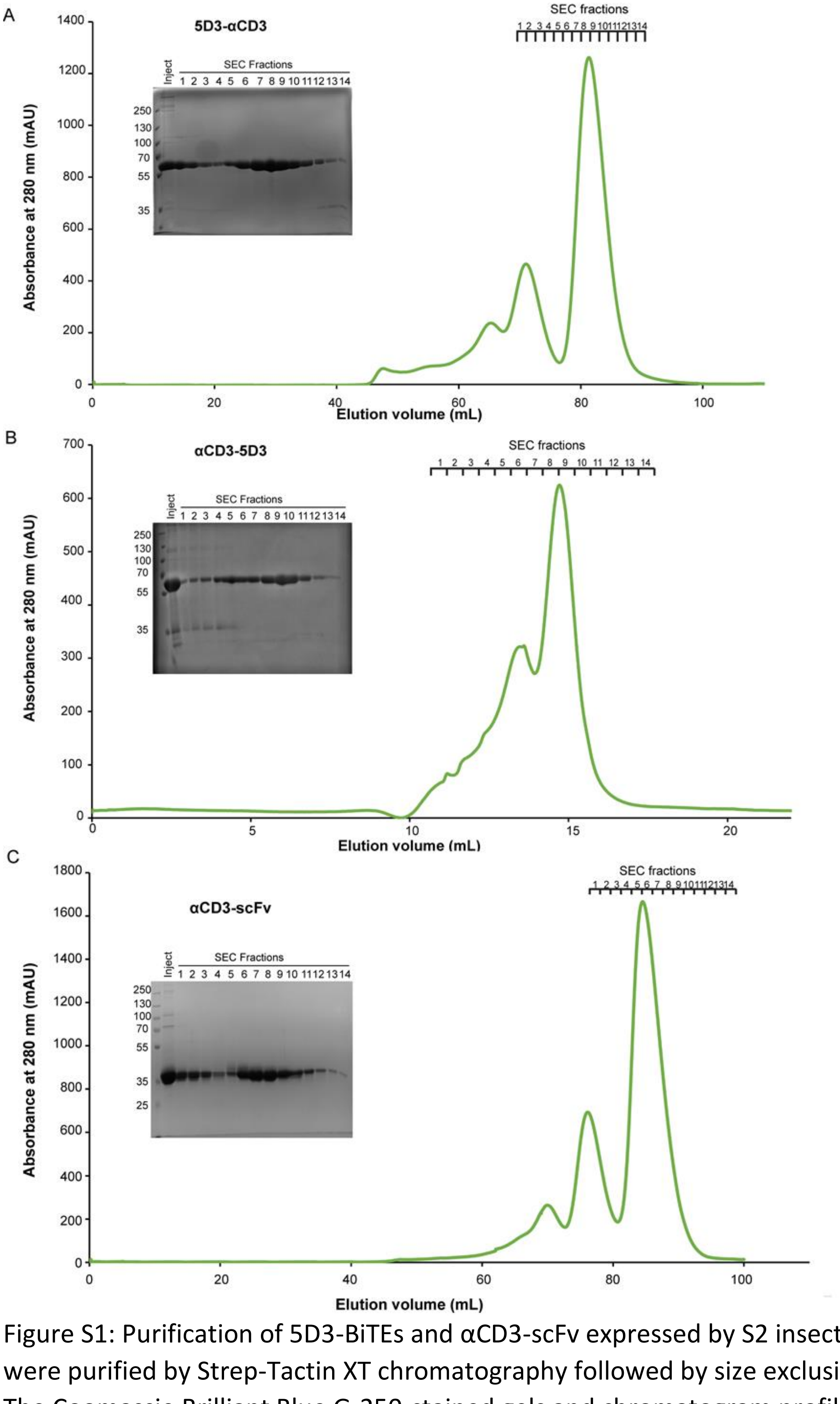

Figure S1: Purification of 5D3-BiTEs and αCD3-scFv expressed by S2 insect cells. The proteins were purified by Strep-Tactin XT chromatography followed by size exclusion chromatography. The Coomassie Brilliant Blue G-250-stained gels and chromatogram profiles are shown for 5D3-αCD3 (A), αCD3-5D3 (B) and αCD3-scFv (C).

Figure S2:

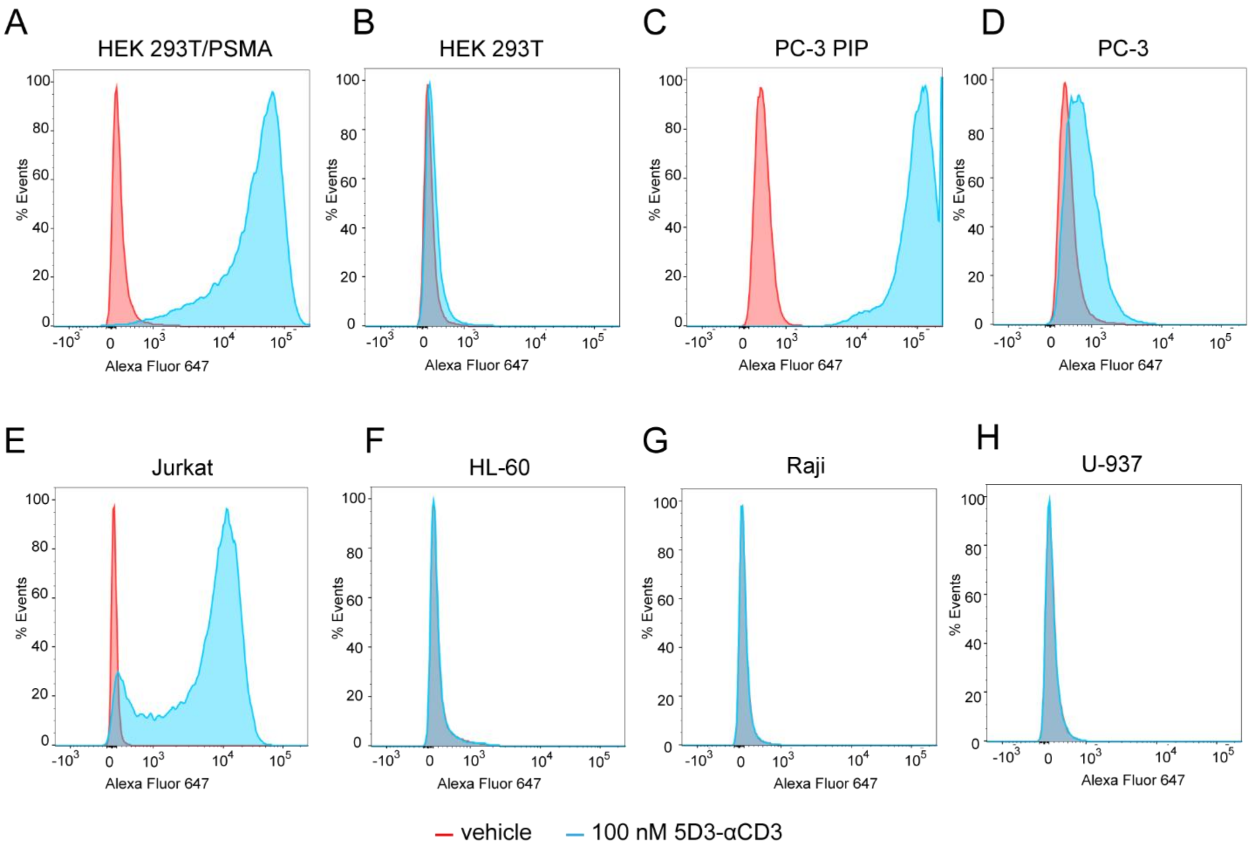

Figure S2: Specificity of 5D3-αCD3 determined by flow cytometry. Cell lines used: HEK 293T/17 overexpressing human PSMA (HEK 293T/PSMA; A), HEK 293T (B), PC-3 PIP overexpressing human PSMA (C), PC-3 (D), CD3-positive Jurkat (E), and CD3-negative HL-60 (F), Raji (G), and U-937 (H) cells. Red-shaded and blue-shaded histograms denote cell populations incubated with either PBS (vehicle) or 100 nM 5D3-αCD3, respectively. Histogram profiles confirm marked specificity of 5D3-αCD3 for PSMA-positive and CD3-positive cell populations.

Figure S3:

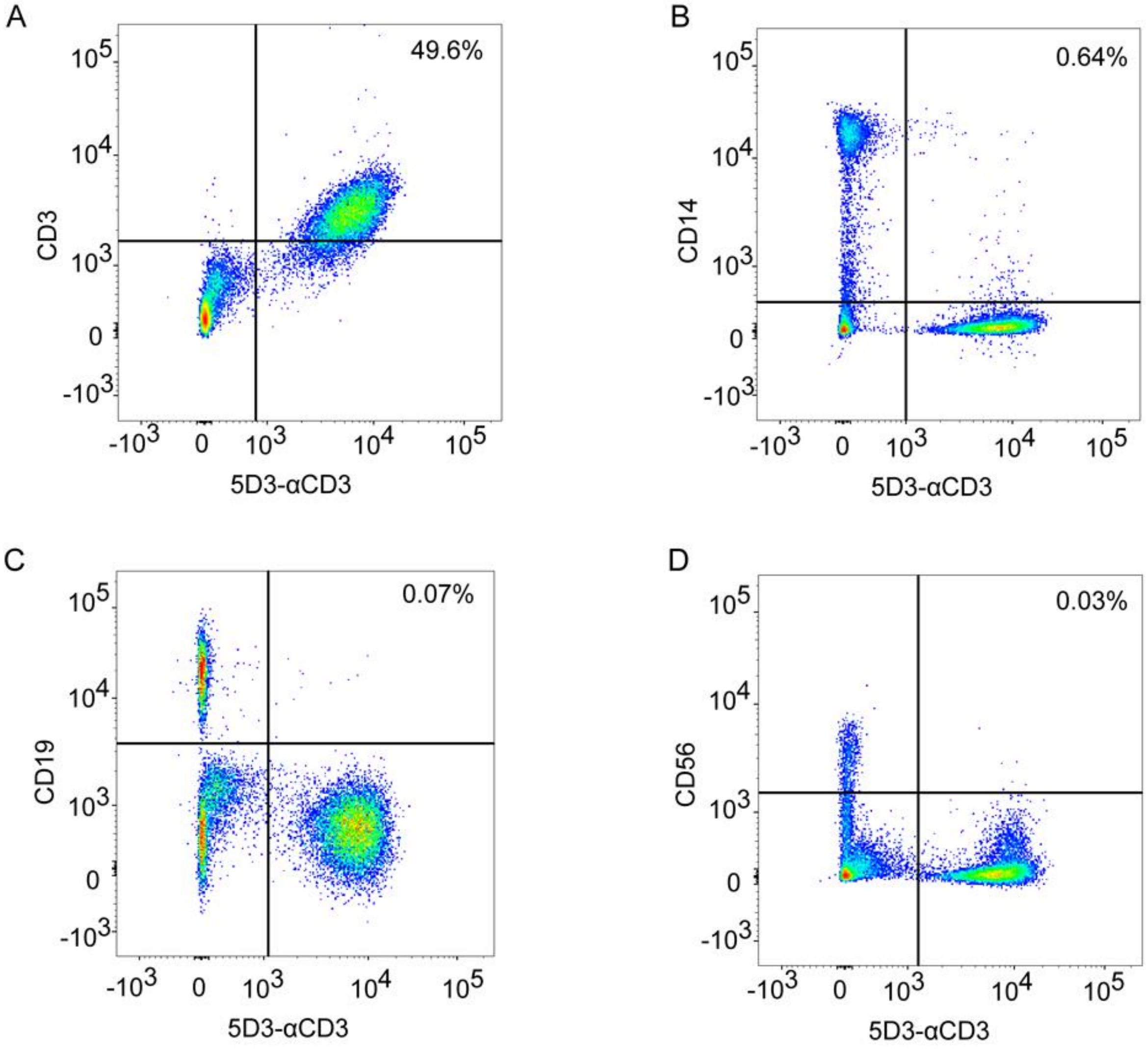

Figure S3: Specificity of 5D3-αCD3 against PBMC cell subsets determined by flow cytometry. PBMCs were doubly stained with the 500 nM BiTE together with antibodies targeting cell-specific surface antigens. CD3 (A), CD14 (B), CD19 (C), and CD56 (D) markers were used to identify populations of T-cells, monocytes, B-cells, and NK-cells, respectively. Only the CD3-positive population corresponding to T-cells reveals simultaneous binding of 5D3-αCD3. The percentage of doubly positive cells is marked in the right upper quadrant of each graph.

Figure S4:

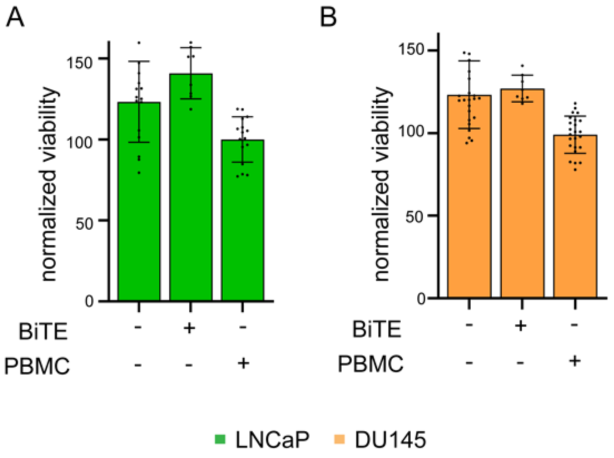

Figure S4: Effect of 5D3-αCD3 on viability of target cells. Target LNCaP (A) and DU145 (B) cells were mixed with either 5 nM 5D3-αCD3 BiTE or PBMCs at the E:T ratio of 3:1. Cell viability was determined using the MTT assay following 48-hour cultivation.

Figure S5:

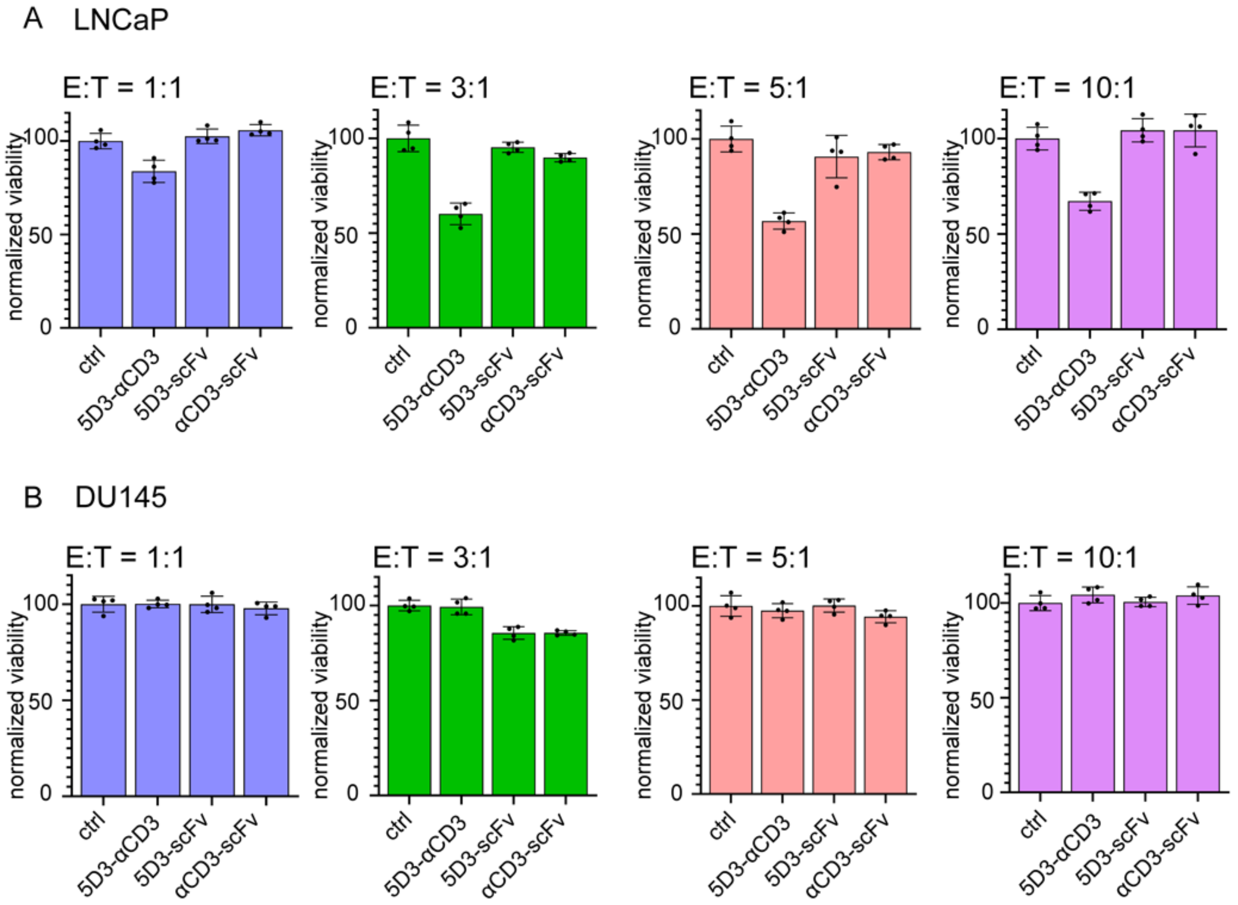

Figure S5: Optimization of the E:T ratio to promote specific killing efficiency of 5D3-αCD3. Co-cultures of PBMCs and LNCaP (A) or DU145 (B) cells (ctrl) in various E:T ratios were mixed with 0.2 nM constructs. Cell viability was measured by MTT assay after 48-hour cultivation of the mixture.
